# Disclosure of HIV status and its impact on the loss in the follow-up of HIV-infected patients on potent anti-retroviral therapy programs in a (post-) conflict setting: A retrospective cohort study from Goma, Democratic Republic of Congo

**DOI:** 10.1371/journal.pone.0171407

**Published:** 2017-02-07

**Authors:** Pierre Zalagile Akilimali, Patou Masika Musumari, Espérance Kashala-Abotnes, Patrick Kalambayi Kayembe, François B. Lepira, Paulin Beya Mutombo, Thorkild Tylleskar, Mapatano Mala Ali

**Affiliations:** 1 Kinshasa School of Public Health, University of Kinshasa, Kinshasa, Democratic Republic of Congo; 2 Department of Global Health and Socio-Epidemiology, Kyoto University School of Public Health, Kyoto, Japan; 3 Department of Global Health and Primary Care, Centre for International Health, University of Bergen, Bergen, Norway; 4 Department of Internal Medicine, University of Kinshasa, Kinshasa, Democratic Republic of Congo; Liverpool School of Tropical Medicine, UNITED KINGDOM

## Abstract

**Background:**

The study aimed to identify the impact of non-disclosure of HIV status on the loss to follow-up (LTFU) of patients receiving anti-retroviral therapy.

**Methodology:**

A historic cohort of HIV patients from 2 major hospitals in Goma, Democratic Republic of Congo was followed from 2004 to 2012. LTFU was defined as not taking an ART refill for a period of 3 months or longer since the last attendance, and had not yet been classified as ‘dead’ or ‘transferred-out’. Kaplan-Meier plots were used to determine the probability of LTFU as a function of time as inclusive of the cohort. The log-rank test was used to compare survival curves based on determinants. Cox proportional hazard modeling was used to measure predictors of LTFU from the time of treatment induction until December 15^th^, 2012 (the end-point).

**Results:**

The median follow-up time was 3.99 years (IQR = 2.33 to 5.59). Seventy percent of patients had shared their HIV status with others (95% CI: 66.3–73.1). The proportion of LTFU was 12% (95%CI: 9.6–14.4). Patients who did not share their HIV status (Adjusted HR 2.28, 95% CI 1.46–2.29), patients who did not live in the city of Goma (Adjusted HR 1.97, 95% CI 1.02–3.77), and those who attained secondary or higher education level (Adjusted HR 1.60, 95% CI 1.02–2.53) had a higher hazard of being LTFU.

**Conclusion:**

This study shows the relationship between the non–disclosure HIV status and LTFU. Healthcare workers in similar settings should pay more attention to clients who have not disclosed their HIV status, and to those living far from health settings where they receive medication.

## Introduction

Although remarkable progress on HIV treatment has been made over the past few decades, HIV infection remains one of the greatest challenges facing the global health community. Access to anti-retroviral therapy (ART) has markedly increased, especially in Sub-Saharan Africa. However, important disparities exist between and within countries; e.g. countries such as the Democratic Republic of Congo (DRC) are still far from reaching universal coverage [1].

In addition to the challenge of increasing access to ART, lost to follow-up (LTFU) as a result of the failure of retention of HIV-infected individuals in treatment programs has been particularly challenging in Sub-Saharan Africa. A range of factors associated with LTFU have been documented, which include—but are not limited to—lack of social support, non-disclosure of HIV status, lower CD4^+^ cell baseline at initiation of treatment, advanced HIV clinical stage (III, IV), regimen type, food insecurity, and drug stock-out [2–4].

Disclosure of HIV status is particularly regarded as a double-edged sword in terms of ART adherence and patient retention in care. There are studies showing the association of HIV status disclosure with better adherence to ART and patient retention in care [5, 6]. One of the postulated mechanisms is that patients who disclose their HIV status to partner(s) or family member(s) are more likely to receive social support, which is a key factor in fostering and maintaining adherence to ART [7]. However, disclosure of HIV status may foster an environment that creates difficulties in patient adherence and retention in care. Some examples exist showing that HIV-infected individuals have lost social support, and have faced discrimination, stigmatization, rejection, and violent reactions following disclosure of their HIV status [8, 9].

Although an extensive literature on HIV status disclosure in Sub-Saharan Africa exists, few studies have addressed its impact on treatment outcomes. Most studies on the association of disclosure with treatment outcomes have examined adherence to ART [5, 6], but very few have investigated its impact on LTFU [10]. Reports on the association of HIV status disclosure with LTFU in conflict or post-conflict settings are remarkably scarce, the only one identified [11], conducted in a post-conflict area in Northern Uganda, only qualitatively linked non-disclosure to LTFU. As far as is known, no report has quantitatively documented the association between non-disclosure and LTFU in a conflict-afflicted setting.

The negative impact of social, economic and political consequences of conflicts on health in general, and on HIV transmission and treatment in particular, has been extensively documented [12]. Conflict or post-conflict settings are often plagued with poverty, breakdown of communities, disruption of social and health systems, and sexual violence [13]. All these circumstances interact in a dynamic way that increases the risk of HIV transmission, negatively affecting patient retention in HIV treatment and care programs. Thus, patients in conflict-afflicted regions may in particular face unique challenges, and their need for social support may be exacerbated. In this regard, disclosure of HIV status can be viewed first as a gateway to accessing social support from the family and the community, and second as a safeguard to limit any adverse consequences, e.g. interruption of HIV treatment, that could result in consequence to a lack of social support. Our study addresses the aforementioned gaps in the literature by investigating the correlates with LTFU, in particular the relationship between LTFU and disclosure of HIV status, among HIV-infected individuals receiving ART in a (post)-conflict setting of the DRC.

## Methods

### Study site and design

A retrospective cohort study on patients was carried out in 2 major hospitals, the Virunga Hospital and the Goma Provincial Referral Hospital (GPRH) in Goma. Goma is a city located in the eastern part of the DRC, where the prevalence of HIV is estimated at 0.9% [14]. The city has seen armed conflict since 1996, and there are still areas where the conflict continues to this day. During the study period, 2 health centers provided HIV care and treatment services, which have been free of charge since 2004.

### Study population and data collection

The study focused on the medical records of patients aged >18 years who were in ART programs between January 2004 and December 2012. All medical records containing information on disclosure HIV status were included in the analysis. Data was collected during the second half of January and the first week of March 2013. Semi-structured and pre-tested questionnaires were used to collect data from the medical records of the patients. This was done by trained data collectors using a pre-designed data extraction tool. All patients with information on disclosure of HIV status in their clinical records were included in the analysis.

To ensure data quality, we trained the supervisors and data collectors on the data collection tools, and were also involved in the pre-testing of the tools as part of this training. The pre-tested tools were checked for consistency and amended as necessary. During data collection, assigned supervisors closely monitored the data collectors both for the validity of the data and ethical issues.

### Treatment, monitoring and information collection

In the DRC, ART began in accordance with guidelines from the World Health Organization (WHO) and the National AIDS Control Program [15, 16]. The first-line treatment comprised Stavudine (D4T) or Zidovudine (AZT), combined with Lamivudine (3TC), and either Nevirapine (NVP) or Efavirenz (EFV). Regimen choice was subject to availability, with a generic fixed-dose combination of D4T, 3TC and NVP being used whenever possible. ART refill appointments are made monthly in the 2 hospitals. Patients CD4 cell and full blood counts (including hemoglobin) were scheduled every 3 months as part of routine follow-up. The characteristics of the treatment programs were also recorded, including procedures in place for tracing patients LTFU. For them, there was an ‘extended follow-up’ in the two hospitals. ‘Extended follow-up’ involved home visits or phone calls using community health agents. Tracing was by telephone contact with patients or their families using information collected at clinic enrollment and home visits. Community health agents had completed high school education and received extra training on HIV/AIDS. They reported the status of each patient to the data clerks of each hospital after each visit.

The following information was collected using a patient’s chart: socio-demographic characteristics (age, gender, marital status and education, residence), disclosure of HIV-infection status (to anyone, at least one other individual) before starting ART, clinical characteristics (type of treatment initiated, nutritional status, alcohol consumption, WHO HIV clinical stage, ART commencement date, date of last contact with the program, mortality date), and biological characteristics (CD4 count and hemoglobin at baseline). Disclosure of HIV status was collected in a clinical interview at the first visit prior starting ART. Nutritional status used Body Mass Index as a proxy assessment. We defined the patient status after extended follow-up as ‘Died’, if a family member, neighbor or community leader reported death of the patient.

### Outcomes

The main outcome variable was LTFU, defined as not taking an ART refill for a period of 3 months or longer from the last attendance for refill, and not yet classified as ‘dead’ or ‘transferred-out’. The date of LTFU was defined from the medical records as that of the last visit to the clinic.

### Statistical analysis

Data were recorded using software Epi Info 7. Data were checked for completeness before entry. A pre-developed Epi Info-based data entry template was designed and given to the data entry clerks. Double entry of the data and thorough cleaning were the other activities used to ensure high quality.

Analyses were carried out using Stata version 12. For continuous variables, medians and interquartile ranges (IQRs) were calculated, proportions and their respective 95% confidence intervals for categorical data. The main outcome variable was LTFU, for which the chi-square test or Fisher´s exact test were used when appropriate.

The incidence rate of LTFU records events per 1000 person-years (p-y) from the date of enrolment. For patients known to have been transferred out, withdrawn or deceased, data were censored at the date of the last appointment or death. Data on patients still in active care at the end of the study period were censored at the date of their last visit to the clinic. Kaplan-Meier curves determined the probability of LTFU as a function of time as inclusion to the cohort. The log-rank test was used to compare survival curves based on determinants. Cox proportional hazard modeling was used to measure predictors of LTFU from treatment induction to the end-point, set at December 15^th^, 2012. Factors associated with LTFU in a bivariate analysis were entered into a Cox regression model to obtain adjusted Hazard ratios and 95% confidence intervals (CI). The following variables were included in the Cox regression model and competing risk models: gender, residence, marital status, alcohol use, disclosure of HIV status, education, and CD4^+^-cell count (<250 cells/μl and ≥250 cells/μl).The proportionality test based on Schoenfeld residuals verified compliance with the assumption of proportionality of risks. All tests were 2-sided and the level of significance set at p<0.05.

### Ethical statement

The original study protocol was approved by the institutional review board ethics committee for research subjects at the Kinshasa University School of Public Health (No App: ESP/CE/034/14 of 27.08.2014). We requested an amendment, which was approved by the institutional review board (No App: ESP/CE/034B/15 of 22.12.2015). Written informed consent was not given by participants for their clinical records to be used in this study. However, patients’ records/information were anonymized and de-identified prior to the analysis.

## Results

### Sample description

**Fig 1:**
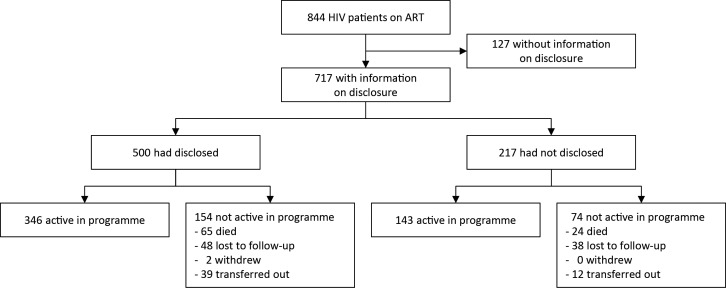
Flow diagram showing the way follow-up of participants was done from January 2004 to December 2012 in 2 different HIV treatment centers at Goma, Democratic Republic of Congo.

A total of 844 HIV patients were enrolled in the HIV program between January 1^st^, 2004 and December 15^th^, 2012. One hundred twenty seven patient records had missing data on HIV disclosure status and were excluded from the analysis, leaving 717 records that met the inclusion criteria for analysis (i.e. 84.2% of the total population from the 2 hospitals (Fig 1).

Overall, the excluded patients were similar to those who were kept in distribution analysis in terms of age (mean 39.8± 9.6 years), gender (64.6% female), residence (92.9% living in Goma), and education (77.5% had none or primary education level).

### Patient characteristics at enrollment

The mean age was 38.2 years (standard deviation [SD] 9.9), and 479 patients (66.8%) were women. At baseline, 115 patients (16.0%) were categorized as full-blown AIDS (WHO stage 4), 399 (55.6%) at stage 3, and 203 (28.3%) at stages 1 or 2. Seventy percent of patients had communicated their HIV status to others (sexual partners or other family members; 95% CI: 66.3–73.1).

Table 1 shows patient characteristics at enrolment and associations with disclosure of HIV status. Male gender and married/cohabiting patients were all significantly associated with disclosure of HIV status before the treatment induction.

**Table 1:** Patient characteristics and disclosure HIV status at enrolment.

Characteristics***	Overall*	HIV status		p
		Disclosure	Non-disclosure	
Age, mean ± SD	38.2 ± 9.9	38.5 ± 10	37.6 ± 9.5	0.275
Age (years)** (n = 717)				0.450
- <median	358(49.9)	245(68.4)	113(31.6)	
- ≥median	359(50.1)	255(71.0)	104(29.0)	
Gender (n = 717)				0.009
- male	238(33.2)	181(76.1)	57(23.9)	
- female	479(66.8)	319(66.6)	160(33.4)	
Education level attained (n = 717)				0.74
- None/ primary	506(70.6)	351(69.4)	155(30.6)	
- Secondary or higher	211(29.4)	149(70.6)	62(29.4)	
Marital status (n = 717)				< 0.001
- married/co-habitating	357(49.8)	276(77.3)	81(22.7)	
- Single	93(13.0)	58(62.4)	35(37.6)	
-Divorced/separated/widowed	267(37.2)	166(62.2)	101(37.8)	
Residence (n = 717)				0.543
- At Goma	664(92.6)	465(70.0)	199(30.0)	
- Outside Goma	53(7.4)	35(66.0)	18(34.0)	
Alcohol (n = 694)				0.702
- yes	233(33.6)	162(69.5)	71(30.5)	
- no	461(66.4)	327(70.9)	134(29.1)	
**Nutritional status at initiation(n = 359)**				0.734
- <18.5 kg/m^2^	68(18.9)	46(67.6)	22(32.4)	
- ≥18.5 kg/m^2^	291(81.1)	203(69.8)	88(30.2)	
Stage of disease (WHO)*(n = 717)*				0.316
- Stage I or II	203(28.3)	134(66.0)	69(34.0)	
- Stage III	399(55.6)	287(71.9)	112(28.1)	
- Stage IV	115(16.0)	79(68.7)	36(31.3)	
Hemoglobin at initiation (n = 606)				0.895
- <10 g/dl	136(22.4)	96(70.6)	40(29.4)	
- ≥10 g/dl	470(77.6)	329(70.0)	141(30.0)	
ART Regimen*(n = 717)*				0.342
- With AZT	246(34.3)	166(67.5)	80(32.5)	
- With d4T	471(65.7)	334(70.9)	137(29.1)	
CD4 (cells/μl) *(n = 717)*				0.254
- <250	561(78.2)	397(70.8)	164(29.2)	
- ≥250	156(21.8)	103(66.0)	53(34.0)	

*percentage calculated for each group

**median age 37.7 years; SD: standard deviation

*** with available data.

### LTFU rate

Of 717 eligible patients, 86 (12%: 95%CI: 9.6–14.4) were LTFU by close of study. Only 142 patients (19.8%) were identified as having been discontinued from care (89 deceased, 51 transferred to other clinics and 2 withdrawn), and were thus not defined as LTFU (refer to S1 Table). A total of 2656.47 p-y were involved in follow-up, with an overall incidence rate of 32.4 (95%CI: 25.5–39.2) patients per 1000 p-y. The mortality rate was 33.5 (95%CI: 26.5–40.5) patients per 1000 p-y (Table 2).

**Table 2:** Multivariate analysis of predictors of LTFU.

Characteristics	n*	Person-years*	Event	LTFU rate	Hazard ratio	
				(per 1000 P-Y)	Crude (95%CI)	Adjusted** (95%CI)
Age (years) *** (n = 717)						
- <mediane	358	1297.8	48	37.0	1.30 (0.85–1.99)	
- ≥mediane	359	1358.7	38	28.0	1	
Gender (n = 717)						
- male	238	776.9	38	48.9	1.85 (1.21–2.83)	1.47 (0.90–2.41)
- female	479	1879.6	48	25.5	1	1
Education level attained (n = 717)						
- none/primary	506	1936.6	50	25.8	1	1
- secondary or higher	211	719.9	36	50.0	1.89 (1.23–2.90)	1.60 (1.02–2.53)
Marital status (n = 717)						
- married/co-habiting	357	1254.5	48	38.3	1.21 (0.68–2.15)	1.06 (0.57–1.96)
- single	93	331.61	15	45.2	0.58 (0.35–0.95)	0.64 (0.37–1.10)
- divorced/separated/widowed	267	1070.4	23	21.5	1	1
Residence (n = 717)						
- In Goma	664	2472.3	75	30.3	1.89 (1.00–3.55)	1.97 (1.02–3.77)
- Outside Goma	53	184.2	11	59.7	1	1
Alcohol (n = 694)						
- yes	233	805.04	38	47.2	1.73 (1.12–2.66)	1.29 (0.82–2.04)
- no	461	1777.6	46	25.9	1	1
Nutritional status at initiation(n = 359)						
- <18.5 Kg/m^2^	68	242.2	9	37.2	1.55 (0.73–3.29)	
- ≥18.5 kg/m^2^	291	1122.0	27	24.1	1	
WHO stage of disease (n = 717)						
- Stage I or II	203	826.5	22	26.6	1	
- Stage III	399	1491.4	49	32.9	1.20 (0.73–1.98)	
- Stage IV	115	338.6	15	44.3	1.50 (0.77–2.88)	
Hemoglobin at initiation (n = 606)						
- <10 g/dl	136	466.4	14	30.0	1.08 (0.60–1.96)	
- ≥ 10g/dl	470	1833.3	49	26.7	1	
Regimen *(n = 717)*						
- With AZT	246	684.9	30	43.8	1	
- With d4T	471	1971.6	56	28.4	1.34 (0.85–2.09)	
CD4 (cells/μl) *(n = 717)*						
- < 250	561	2044.9	75	36.7	2.00 (1.06–3.77)	1.81 (0.95–3.42)
- ≥ 250	156	611.6	11	18.0	1	1
Disclosure HIV status (n = 717)						
- no	217	713.3	38	53.3	2.03 (1.32–3.11)	2.28 (1.46–2.29)
- yes	500	1943.2	48	24.7	1	1
Overall	**717**	**2656.5**	**86**	32.4		

*calculated with available data

**adjusted with associated factors with LTFU by bivariate analysis with p-value ≤0.05

***median age 37.7 years.

### Predictors of LTFU among HIV-infected patients on ART

**Fig 2:**
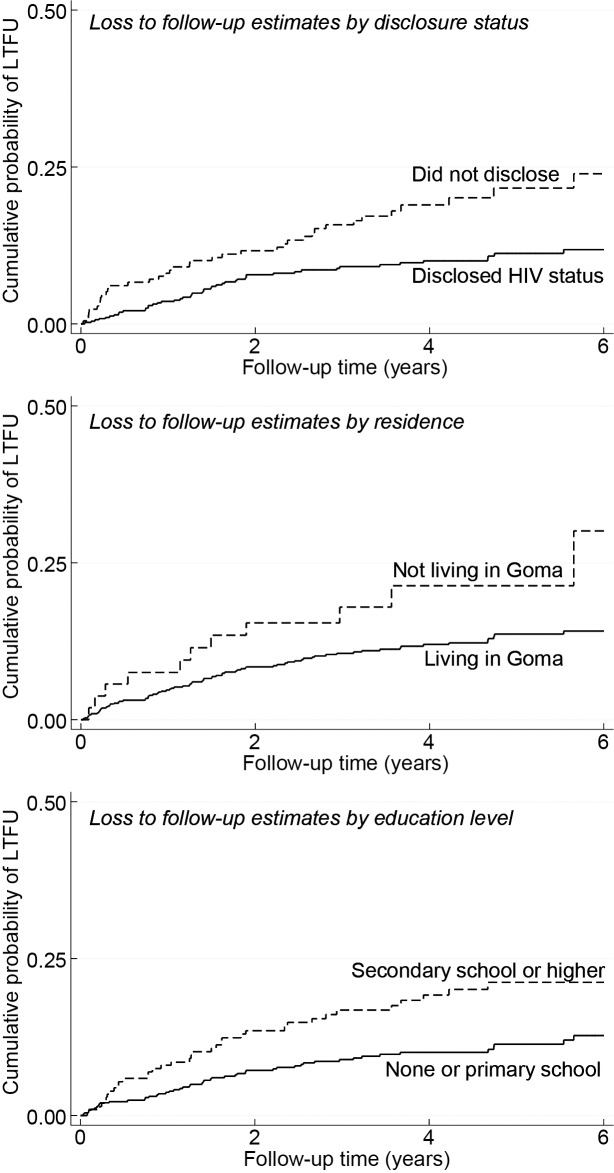
Cumulative incidence of LTFU by residence, disclosure status and education level.

Patients who did not share their HIV status had a higher hazard of being LTFU than those who did (adjusted HR 2.28, 95% CI 1.46–2.29). Patients not living in the city of Goma had a higher risk of being LTFU compared with those living in Goma (adjusted HR 1.97, 95% CI 1.02–3.77). Last, patients who attained secondary or higher education level had a higher risk of being LTFU compared with those of a lower education level (adjusted HR 1.60, 95% CI 1.02–2.53) (Table 2 and Fig 2).

## Discussion

To our knowledge, this is the first study to quantitatively document the association of HIV status disclosure and LTFU in a post-conflict setting in Sub-Saharan Africa. We found that 70% of HIV-infected patients had shared their HIV status with others at the time they were enrolled in HIV care, and that non-disclosure of HIV status was associated with a greater hazard of being LTFU.

The findings are in line with previous research indicating that, despite the complex nature of the disclosure process, HIV prevalence in Sub-Saharan Africa remains high. For example, a review of social and gender context of HIV disclosure in Sub-Saharan Africa found that most reports gave rates of disclosure >74% [17]. Similar rates have been reported in Mali, Burkina Faso, and Uganda [18, 19]. A recent study in the DRC found that 77.1% of patients receiving ART had disclosed their HIV status [20]. However, studies focusing on HIV status disclosure in conflict and/or post-conflicts settings remain lacking. The relationship between non-disclosure of HIV status and increased risk of LTFU found in our study may potentially be mediated by the effect of social support. Although disclosure of HIV status may have dual effects in terms of accessing social support, it appears from the literature that the benefits associated with disclosure overwhelmingly outweigh potential adverse effects, such as loss of social support, stigma and discrimination [8, 9, 17, 21, 22]. Thus, patients who do not disclose their status are often less likely to receive social support and perform poorly in terms of achieving optimum levels of adherence and retention in care. In the context of a post-conflict setting, where basic social infrastructures might be in disarray and poverty often prevails, social support from the family and community is vital in fostering engagement of a patient in HIV treatment and care. Social support comes in multiple forms, including financial support, physical support (help in care), and psychological support [23], all of which can help patients cope with the day-to-day challenges of living with HIV. For example, financial support can alleviate transportation costs needed for some patients to make clinical visits [21]. Our study patients who did not disclose their status might have lacked such a support, and therefore were at greater risk of defaulting on HIV treatment. It is important to emphasize, particularly in a post-conflict setting, that insecurity rather than a lack of social support could be the main factor for non-attendance at clinics. Therefore, there is need for studies to disentangle the possible pathways linking non-disclosure with LTFU. Such studies should explore in particular the possible mediating effect of social support in the relationship between HIV status disclosure and LTFU, as also the direct impact of insecurity on LTFU.

Our finding that patient living outside Goma had a higher risk of LTFU puts in context the aforementioned possibility that lack of social support could mediate the association between non-disclosure and LTFU; however, the direct impact of insecurity may have an effect on patient attendance at clinics. Armed conflict in the city has led the displacement of many patients to places far away from their health facilities, with consequences in terms of the cost incurred for accessing the health facility. This means that patients in need of, but lacking, social support are likely to default on treatment. Many studies have reported that travel time to the clinics and its associated opportunity cost (in terms of financial cost or time that could be allocated to something else) are important barriers to patient adherence to ART and retention in care [24–26]. Travelling long distances to reach the health facility also means taking care for one’s security, especially when travelling across dangerous areas. Thus, many patients might decide to interrupt their anti-retroviral medication. Policy makers, government and international agencies should quickly resolve this issue through innovative strategies that can either reduce the cost or the distance to the health facility. In Mozambique, the cost of travel has been substantially reduced by patients living in the same area creating organized groups who took turns for visiting clinics to collect medication for all the group members [27].

While many studies have found either no association between level of education and LTFU [28–30] or low to poor educational level being associated with LTFU [31–33]. We found that individuals with higher level of education were at greater risk of LTFU. With regard to the context of Goma as an area of conflict, we assume that most of patients have migrated to stable areas. Overall, research on the relationship between education and migration in Africa gives mixed results, with some research showing that individuals with higher education are more likely to stay at home, whereas others indicate that those with more education are more likely to migrate [34]. The relationship between migration and education could depend on the context, the type of migration and the reason for it. Increasing income, education and access to information and networks generally increase peoples’ abilities and aspiration to migrate. In areas with insecurity, it is not surprising that individuals with a high level of education are more likely to migrate, and they more often have access to resources (of many forms, such as economic and social). Therefore they can move to more stable areas than those with fewer resources [35]. We postulate that individuals with higher level of education might have more access to economic resources, and that the particular context of insecurity in the region could have prompted them to migrate to more stable areas. Similar studies conducted in other post-conflict areas could confirm whether this phenomenon is global or specific to the context of the DRC.

The association of non-disclosure with LTFU shown herein also highlights the need for a thorough investigation to understand the contextual and social factors that shape disclosure and non-disclosure of HIV status in these settings. While many patients in Sub-Saharan Africa disclose their HIV status, a considerable proportion chose not to disclose for a number of reasons. For example, stigma (internalized or experienced), discrimination and fear of divorce or abandonment have all been cited as important barriers to HIV status disclosure [36–39]. Interventions and programs should encourage HIV status disclosure; however, care needs to be taken to avoid adverse effects that can result from disclosure in a community where HIV-related stigma prevails.

This study has several limitations, the main one being that it is of a partially retrospective design and the fact that it has relied heavily on patients' charts, leading to missing data and possible information bias. Second, a possibility of misclassification might be due to the sensitive nature of the disclosure of HIV status; individuals may incorrectly report on having disclosed their HIV status to others. Third, HIV status disclosure was reported as a dichotomous variable only at enrolment, yet it is a process that evolves with time. The date of disclosure was frequently missing, making it impossible to study this variable as time-dependent information in survival analyses. The research team could not ascertain the true outcomes of the patients who were documented as being LTFU due to insecurity in the region over that period of time. Lastly, living outside Goma has been recognized as a factor associated factor with LTFU; however, we did not have data that allowed us to assess properly this association. Nevertheless, this study has the advantage of being among the few to document a relationship between LTFU and disclosure of HIV status in Africa in general, and particularly in a context of conflict and post-conflict. The results might help researchers, healthcare workers, and other stakeholders involved in HIV treatment and care to understand factors associated with LTFU in conflict-affected settings.

## Conclusion

This study reports a strong effect of non–disclosure of HIV status on LTFU. Healthcare workers in similar settings should pay more attention to clients who did not disclose their HIV status, and to patients living far from the city where care is given during the pre-ART phase. More targeted counseling and follow-up is needed. Further studies should also look at the effect of non-disclosure of HIV status on other outcomes, such as immunological and nutritional responses.

## Supporting information

S1 Table Outcomes of patients included in the study. (DOCX)
