# Supplementary material for: Disclosure of HIV status and its impact on the loss in the follow-up of HIV-infected patients on potent anti-retroviral therapy programs in a (post-) conflict setting: A retrospective cohort study from Goma, Democratic Republic of Congo
Source: PLoS One. 2017 Feb 7;12(2):e0171407. doi: 10.1371/journal.pone.0171407 (PMC5295697; doi:10.1371/journal.pone.0171407)
Supplement: S1 Table — (DOCX) [file pone.0171407.s001.docx]

Table S1: Outcomes of Patients included in the study

| Outcomes | frequency | % |
| --- | --- | --- |
| Alive | 489 | 68.2 |
| deceased | 89 | 12.4 |
| Withdrew from care | 2 | 0.3 |
| Loss to follow-up | 86 | 12.0 |
| Transferred | 51 | 7.1 |
| Total | 717 | 100 |
